# Impairment characteristics of static balance and plantar load distribution of patients undergoing tibial cortex transverse distraction for diabetic foot ulcers

**DOI:** 10.1186/s13018-022-03042-3

**Published:** 2022-03-18

**Authors:** Zhi-Qiang Fan, De-Wu Liu

**Affiliations:** 1 Department of Orthopaedic Surgery, Jiangxi Provincial People’s Hospital Affiliated To Nanchang University, 152 Ai Guo Road, Nanchang, 330006 Jiangxi People’s Republic of China; 2 Institute of Burn, The First Affiliated Hospital of Nanchang University, Nanchang, Jiangxi China

**Keywords:** Diabetic foot ulcers, Tibial cortex transverse distraction, Static balance, plantar load, Center of pressure

## Abstract

**Objective:**

Tibial cortex transverse distraction (TCTD) has been recently reported for the treatment of diabetic foot ulcers. Herein, we explored the characteristics of the impairments in static balance and plantar load distribution in patients.

**Methods:**

We performed a retrospective study of 21 patients with diabetic foot ulcers who underwent TCTD, who were regularly followed up for > 1 year after surgery, and 20 healthy individuals (control group). A pressure platform was used to assess the standing balance functions of the lower extremities and the plantar load distribution.

**Results:**

One patient underwent amputation because of severe infection. In patient group, center of pressure (COP) ellipse sway area, COP path length and angle *θ* were all larger, compared with those of control group (250.15 ± 98.36 mm^2^ vs. 135.67 ± 53.21 mm^2^, 145.15 ± 67.43 mm vs. 78.47 ± 34.15 mm, 39.75 ± 17.61° vs. 22.17 ± 14.15°), with statistically significant differences (*P* < 0.01). The average plantar load and backfoot load of the unaffected side was significantly larger than that of the affected side (58.4 ± 5.5% vs. 41.6 ± 5.5%, 45.3 ± 6.4% vs. 36.5 ± 5.6%), but they were similar for the two feet of members of the control group.

**Conclusions:**

Although TCTD may represent an appropriate method for the treatment of diabetic foot ulcers, postoperative impairments in static balance and plantar load distribution remain in the long term. These potential long-term problems should be taken into account in further rehabilitation planning.

*Type of study/level of evidence*: Therapeutic III.

## Introduction

The "diabetic foot" is a serious chronic complication of diabetes mellitus that can lead to disability and death and is a major cause of non-traumatic amputation [1]. The incidence of diabetic foot is approximately 8.0% in hospitalized patients with diabetes in China, and most of these patients demonstrate ulceration and infection [1, 2]. The treatment of diabetic foot is difficult and costly, and places huge financial burdens on families and society. The pathogenesis of diabetic foot mainly involves peripheral nerve lesions and peripheral artery diseases (PAD) [3, 4]. Current pharmacotherapy for diabetic foot has made some progress, the main therapies are still focused on surgery treatments [5, 6]. The key challenge of diabetic foot therapy is to restore blood flow of the affected limb and to improve microcirculation and oxygen supply to the ulcer and adjacent tissues. [6, 7]

Vascular lesions of the diabetic foot are typically treated by vascular surgery, which may involve arterial bypass transplantation, endovascular shaping and/or stem cell transplantation [7–9]. Although the treatments used can vary, the net effect is often very limited and some patients still require amputation [8, 9]. Tibial cortex transverse distraction (TCTD) has been recently reported as an alternative means of treating diabetic foot ulcers [10, 11]. TCTD was designed according to the “tension-stress rule” of Ilizarov technology and is the application of microcirculation reconstruction in the post-Ilizarov era. By applying transverse, slow and continuous traction to the tibial cadre bone window, the regeneration of surrounding tissue is stimulated, and the ischemic necrosis of the tissues and neuropathy are reduced, thereby permitting the healing of diabetic foot lesions.

In previous studies, the Zebris pressure platform was used for evaluate percentage of body weight distribution, static and dynamic posture and gait and balance [12, 13]. Morasiewicz et al. used this Zebris pressure platform to analyse the balance and body weight distribution on the lower extremities in patients after ankle arthrodesis with Ilizarov fixation and internal fixation [12–14]. They analyzed in depth the comparison of external fixation stent and internal fixation in multiple studies, on the specific impact of weight bearing and balance of lower limbs, and provided a research basis for further treatment. We learnt that this evaluation technique was a very reliable and scientific method, and can also be used in the evaluation of patients with diabetic foot ulcers who underwent TCTD.

Although TCTD represents a potentially viable alternative method for the treatment of diabetic foot ulcers, the postoperative function of affected lower limb has not been properly characterized. We propose hypotheses that the standing balance and plantar load distribution remain impaired after TCTD procedure. In the present study, we used a Zebris pressure platform to compare the standing balance function of the lower extremities and the plantar load distribution of patients who underwent TCTD and those of healthy age-matched individuals. The results of this study should provide a reference for the future design of rehabilitation strategies.

## Materials and methods

### Patient and control groups

This study was conducted in accordance with the principles of the Declaration of Helsinki and its amendments for Human Research. Written informed consent was obtained from all participants and their rights to privacy were respected. We performed a retrospective analysis of data collected during the visits of patients to our clinic, and consequently the institutional review board exempted the study from the necessity for ethics approval. The participants comprised consecutive patients who had diabetic foot ulcers and underwent TCTD in combination with debridement and vacuum sealing drainage (VSD) between March 2017 and March 2019 at the First Affiliated Hospital of Nanchang University, Jiangxi Provincial People’s Hospital Affiliated to Nanchang University, China. The criteria for inclusion include: (1) patients diagnosed with diabetic foot ulcers and undergoing TCTD; (2) no trauma or tumor around the ulcer zone (3) detailed medical history data; (4) long-term follow-up results. The criteria for exclusion include: (1) no detailed medical history was available; (2) patients did not cooperate with routine follow-up or visits; (3) combined other serious diseases that affect treatment. The participants of control group were healthy individuals who mainly were the patients’ relatives. We have de-identified the participant details such that their identities cannot be ascertained in any way. The reporting of this study conforms to the STROBE guidelines.

### Preoperative management

After admission, the patients underwent a routine preoperative examination. We evaluated their general condition, adjusted the blood glucose level to less than 10 mmol/L, and performed vascular imaging and ultrasonography to assess the patency of the vessels in the affected limb. If there was necrotic tissue or serious infection, the necrotic part of foot was completely removed. Bacterial culture and drug sensitivity test of wound secretion was performed. The wound of foot was covered with VSD, and antibiotics were applied according to the results of drug sensitivity test.

### TCTD technique

We have described the surgical procedure in detail in a previous study [11]. After the induction of general or lumbar anesthesia, the osteotomy area was selected as the medial tibial cortex, approximately 10–20 cm distal to the knee joint. After positioning, two half-nails were used to fix the external fixation frame at the proximal and distal ends of the tibial osteotomy area, and then two half-nails were placed in the osteotomy area. An incision of 3 cm was made at the distal and proximal ends of the medial tibial osteotomy area, the subcutaneous tissue was separated and the medial aspect of the tibia was exposed. After drilling, a bone flap was raised from the tibial shaft using a bone knife. After determining the complete free bone flap, we completed the assembly of the external fixation device and sutured the incision. For wounds with a large infected area and substantial tissue necrosis, we performed intraoperative debridement and applied VSD.

### Postoperative management

After surgery, appropriate antibiotics, pain-relieving medication and hypoglycaemic medication were continued. The dressings of wound were changed and the pin-sites were disinfected regularly. For the 3 days immediately following surgery, analgesic administration was stopped. TCTD was commenced between postoperative days 3 and 5. We carried out 1 mm distraction every day for 14 days in total. Then, we reverse-moved the bone mass by 1 mm per day. Thus, approximately 28–30 days were needed to complete the total distraction. Subsequently, X-ray images were routinely obtained monthly to monitor bone healing, and once the bone window had healed, the external fixation frame was removed.

Foot skin temperature, pain visual analogue score (VAS), ankle brachial index (ABI) and wound healing were closely monitored during treatment. Computed tomography angiography (CTA) was conducted to analyze the resulting vascular hyperplasia.

### Evaluation of static balance and plantar load distribution

We used a Zebris pressure platform (Zebris Medical GmbH, Isny im Allgäu, Germany) to evaluate the static balance and plantar load distribution of the participants. Before commencing these tests, we explain the test process to the participants to facilitate their cooperation and the collection of more accurate data. During the measurements, the participants stood with their eyes open, bare feet, and in a relaxed pose. Their arms were placed lateral to their bodies, with their shoulders placed the same distance apart, and they were instructed to look at an object 2 m directly in front of them. This is the standard standing test posture. The average value of each parameter is processed by the software for further analysis.

**Fig. 1:**
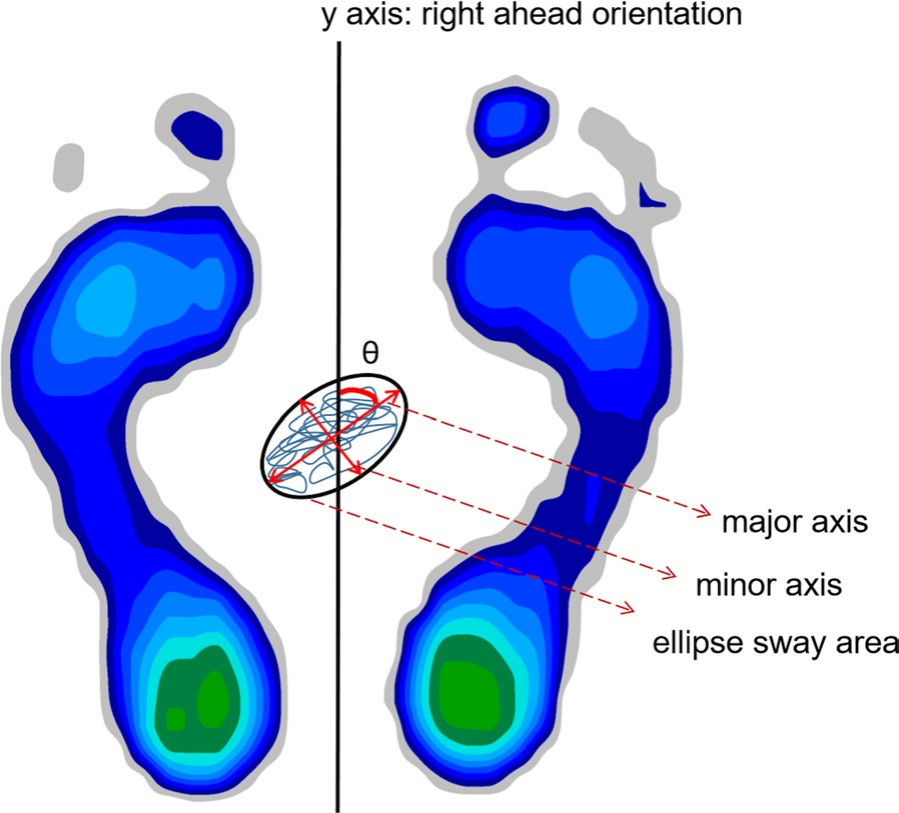
Schematic of parameters included in this study with Zebris pressure platform evaluating static balance and plantar load distribution

The static balance parameters that were assessed included center of pressure (COP) ellipse sway area, COP path length and angle *θ* between y and major axis of ellipse sway area (Fig. 1). [15, 16] The larger value of COP ellipse sway area and COP path length were, the worse was the participant’s static balance. Large *θ* angles indicated that the instability was principally lateral, whereas small *θ* angles indicated that the instability was principally anterior–posterior. The load distribution of the left and right feet, and the forefoot and backfoot of the subjects were also evaluated. The mean values for each parameter were calculated and further analyzed.

### Statistical analyses

SPSS version 19.0 (IBM Corp, Chicago, IL, USA) was used to calculate the mean ± SD of continuous data. Student’s t-test was used to compare preoperative and postoperative data within and between groups. Statistical significance was set at *P* < 0.05.

## Results

**Fig. 2:**
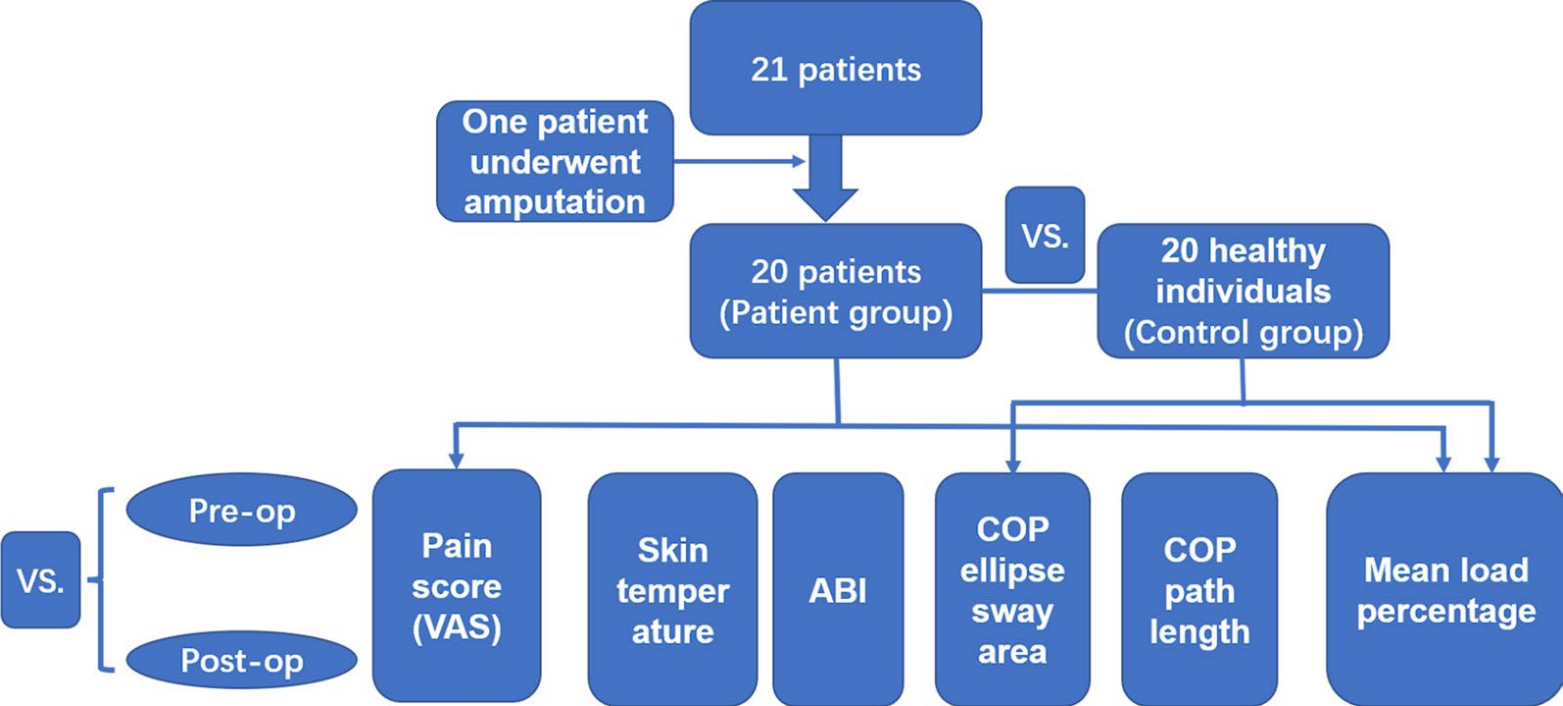
The flow chart of this study

The flow chart of this study is shown in Fig. 2. Twenty-one consecutive patients (15 men and 6 women) who had been diagnosed with diabetic foot ulcers were recruited. Their median age was 52 years (range 42–65 years). Their median period of time that had elapsed since they were first diagnosed with diabetic was 14.5 years (range 6.4–28 years). Twelve participants had ulcers of their left foot and nine had ulcers of their right foot. We classified the foot ulcers according to their Wagner stage, and found that four had stage 4 ulcers, 14 had stage 3 ulcers, and two had stage 2 ulcers. CTA of both lower extremities identified arterial occlusion distal to the knee joint or varying degrees of stenosis, and other causes of arterial lesions could be excluded. In addition, a total of 20 healthy individuals were recruited as the control group (15 males and 5 female), who had a median age of 51 years (range 40–64 years). There were no significant difference of the age and sex between two groups (*P* > 0.05). We also compared the BMI of patients and control group, and there was no significant difference between them (*P* > 0.05).

**Fig. 3:**
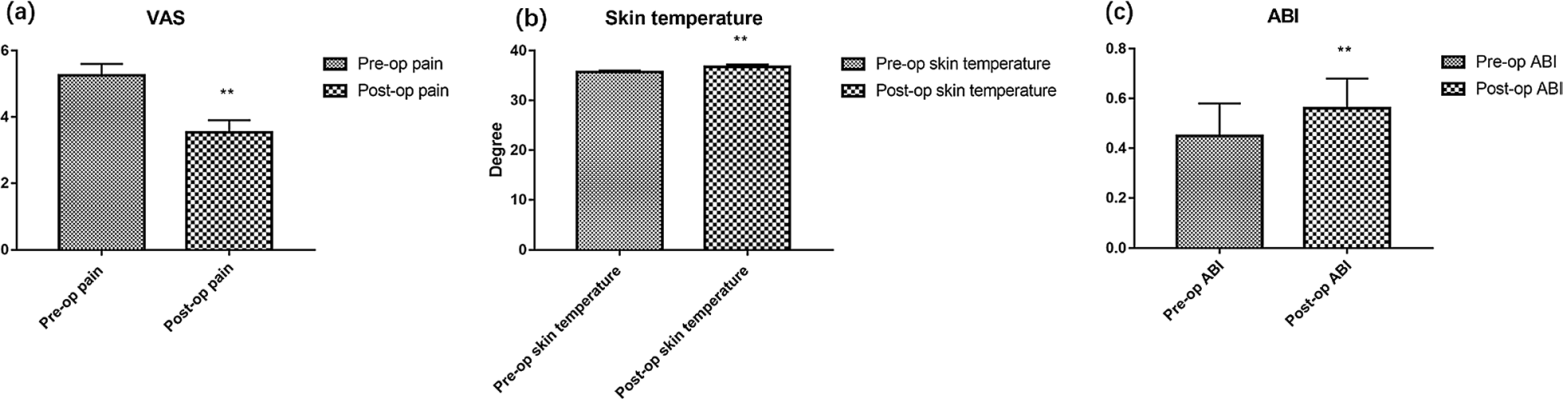
Comparisons of pain (**a**), skin temperature (**b**) and ABI (**c**) between preoperative and postoperative values in patient group (**P < 0.01)

We followed the patient group for a mean 14.5 months (range 13–23 months). One of the participants underwent amputation because of a serious infection. but none experienced other complications, such as tibia fractures during this period. The ulcers of the other 20 patients had healed by 7.1 ± 3.3 weeks following surgery. Postoperative pain, assessed using a VAS, was significantly lower than preoperative pain (preoperative 5.25 ± 0.35 vs. postoperative 3.53 ± 0.37, *P* < 0.01, Fig. 3a). The skin temperature of the participants after surgery was significantly higher than before (preoperative 35.65 ± 0.34 vs. postoperative 36.73 ± 0.52, *P* < 0.01, Fig. 3b). Postoperative ABI (0.56 ± 0.12) was also significantly higher than preoperative ABI (0.45 ± 0.13) (*P* = 0.008, Fig. 3c). CTA showed the formation of collateral circulation, with the superficial artery of the lower extremities becoming thicker and forming a net.

**Fig. 4:**
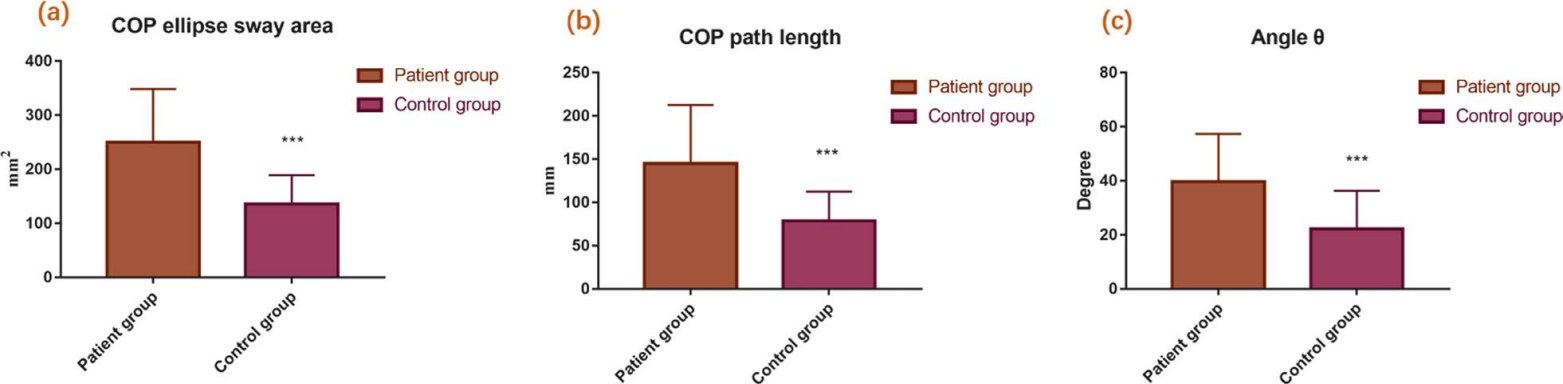
Comparisons of COP ellipse sway area (**a**), COP path length (**b**) and angle *θ* (**c**) of the participants between patient group and control group (***P < 0.001)

**Fig. 5:**
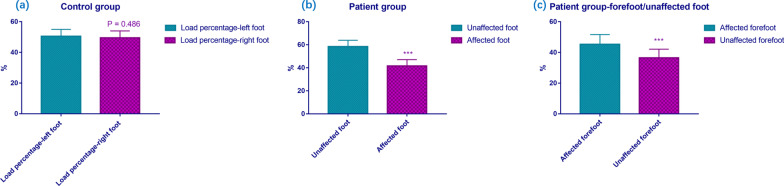
Comparisons of average load percentage in patient group and control group. **a** There was no significant difference of load percentage between two feet in the control group. **b** The average load percentage of the unaffected foot was significantly larger than that of the affected foot in the patient group. **c** The mean load percentage of the affected forefoot was significantly larger than that of the unaffected forefoot in the patient group (***P < 0.001)

**Fig. 6:**
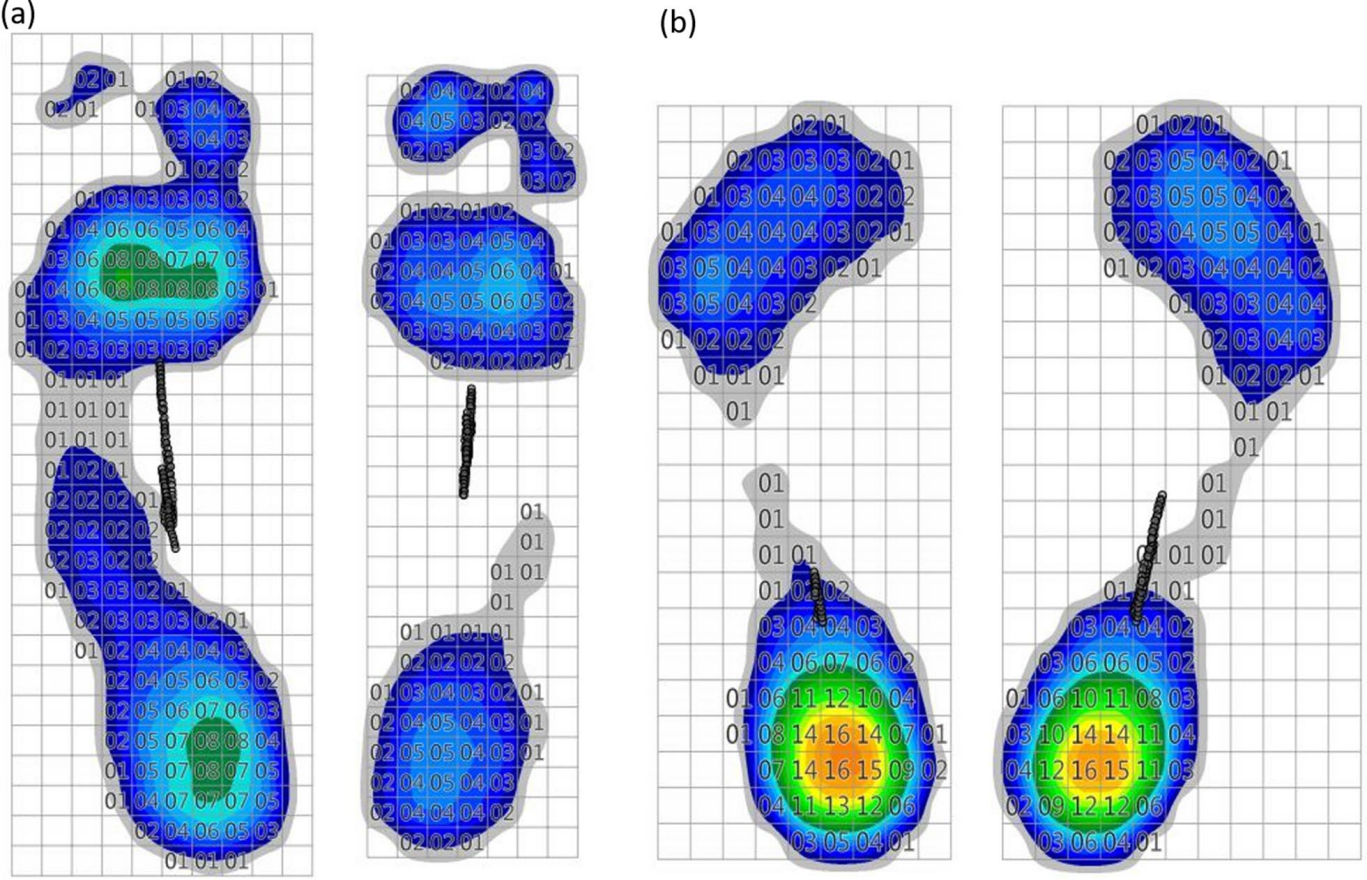
The plantar pressure map of a patient (right affected foot) and a healthy individual, which demonstrated the unaffected foot of patient bore more pressure, and the load percentage of the backfoot of the affected decreased (**a**). In the healthy individual, the pressure distribution of two feet is coincident, and the load percentage of the backfoot is larger than that of the forefoot (**b**)

**Fig. 7:**
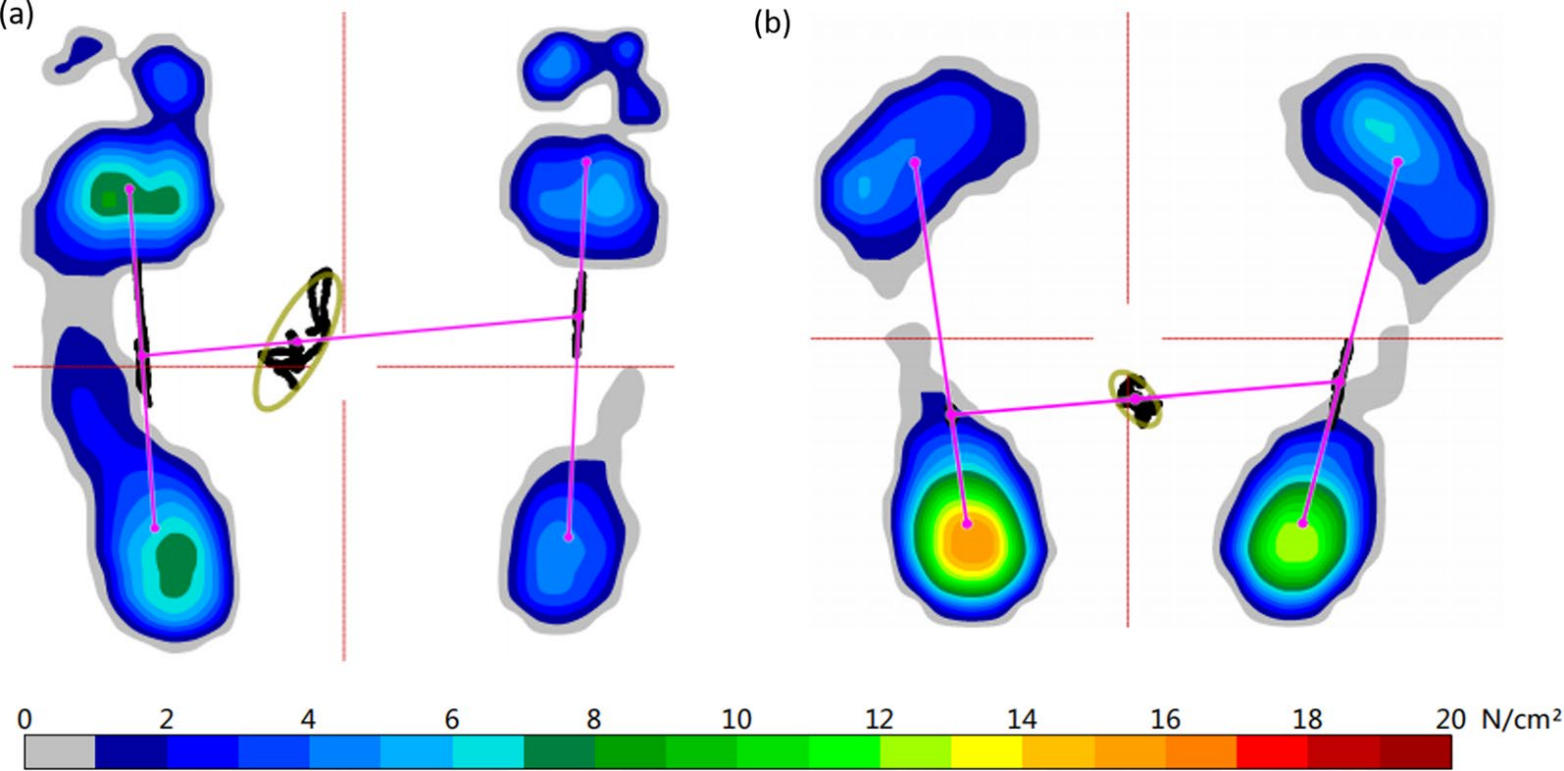
The balance factors of a patient (right affected foot) and a healthy individual, which indicated that the COP ellipse sway area, COP path length and angle *θ* are all larger

The 20 patients who did not undergo amputation were evaluated using a Zebris pressure platform. The COP ellipse sway area of the patient group was 250.15 ± 98.36 mm^2^, and that of the control group was 135.67 ± 53.21 mm^2^ (*P* < 0.001, Fig. 4a). The COP path length of patient group was 145.15 ± 67.43 mm, and that of the control group was 78.47 ± 34.15 mm (*P* < 0.001, Fig. 4b). Thus, there was significant impairment of the static balance function of the patient group. The angle *θ* of the patient group was 39.75 ± 17.61°, and that of the control group was 22.17 ± 14.15° (*P* = 0.001, Fig. 4c). In the control group, the average load percentage of the left foot was 50.5 ± 4.5%, and that of the right foot was 49.5 ± 4.5%. There was no significant difference between two feet (*P* = 0.486, Fig. 5a). In contrast, in the patient group, the average load percentage of the unaffected foot was 58.4 ± 5.5%, and that of the affected foot was 41.6 ± 5.5%. There was significant difference between two feet (*P* < 0.001, Fig. 5b). The mean load percentage of the affected forefoot was 45.3 ± 6.4% (forefoot/unaffected foot), and that of the unaffected forefoot was 36.5 ± 5.6%. There was significant difference between two forefeet (*P* < 0.001, Fig. 5c). Accordingly, the difference in the mean load percentage between the two backfoot was also significant (*P* < 0.001). The comparisons of the clinical data of the participants between the control group and the patient group were concluded in Table 1. Figure 6 shows the plantar pressure map of a patient (right affected foot) and a healthy individual, which demonstrated the unaffected foot of patient bore more pressure, and the load percentage of the backfoot of the affected decreased (Fig. 6a). In the healthy individual, the pressure distribution of two feet is coincident, and the load percentage of the backfoot is larger than that of the forefoot (Fig. 6b). Figure 7 shows the balance factors of a patient (right affected foot) and a healthy individual, which indicated that the COP ellipse sway area, COP path length and angle *θ* are all larger.

**Table 1:** Comparison of the clinical data of the participants between the control group and the patient group

Item		Patient group			Control group	*P*
Gender	Men	15			15	> 0.05
	Woman	5			5	
	Amputation (exclude)	1				
Age-year	Median	52			51	> 0.05
	Range	42–65			40–64	
Foot	Left	12				
	Right	9				
BMI		23.5 ± 4.5			23.7 ± 6.5	> 0.05
VAS	Preoperative	5.25 ± 0.35				< 0.01
	Postoperative	3.53 ± 0.37				
Skin temperature	Preoperative	35.65 ± 0.34				
	Postoperative	36.73 ± 0.52				< 0.01
ABI	Preoperative	0.45 ± 0.13				
	Postoperative	0.56 ± 0.12				0.008
COP ellipse sway area-mm^2^		250.15 ± 98.36			135.67 ± 53.21	< 0.001
COP path length-mm		145.15 ± 67.43			78.47 ± 34.15	< 0.001
Angle θ-°		39.75 ± 17.61			22.17 ± 14.15	0.001
Average load percentage-%	Unaffected foot	58.4 ± 5.5^a^		Left foot	50.5 ± 4.5^c^	a versus b < 0.001
	Affected foot	41.6 ± 5.5^b^		Right foot	49.5 ± 4.5^d^	c versus d 0.486
Mean load percentage-%	Affected forefoot	45.3 ± 6.4				< 0.001
	Unaffected forefoot	36.5 ± 5.6				

^a^Average load percentage of unaffected foot in patient group

^b^Average load percentage of affected foot in patient group

^c^Average load percentage of left foot in control group

^d^Average load percentage of right foot in control group

## Discussion

The "diabetic foot" is a serious complication of diabetes mellitus. The condition is complex and the treatment methods used are diverse, and often not particularly effective. The TCTD procedure can stimulate the regeneration of peripheral tissue by applying transverse, slow and continuous traction to the bone window of the tibial cadre [11, 15]. This promotes the regeneration of the bone window and the distal microvascular network, ameliorating the ischemia and hypoxia of distal tissue, thereby reducing the ischemic necrosis and neuropathy associated with diabetic foot and permitting healing. [11, 15]

In the present study, one participant underwent amputation because of severe infection, but the ulcers of the other 20 patients had healed by a mean 7 weeks following surgery. Their pain VAS score was significantly lower postoperatively than preoperatively. Therefore, TCTD in combination with debridement and VSD is clinically efficacious.

Standing is the most basic activity in daily life, and therefore its importance is self-evident [17, 18]. When healthy people stand still, the weight distributions of their lower limbs are basically symmetrical. However, patients with diabetic foot ulcers show lower muscle strength in the affected limb, which may be associated with muscle atrophy, and this contributes to the asymmetric load distribution on the lower limbs. The present analysis also suggests that there is significant posture control instability in patients with diabetic foot ulcers. The *θ* angle of the patient group was significantly larger than that of the control group, which implies that the principal instability is in the lateral plane. Therefore, static standing balance training should focus on increasing stability in the lateral plane.

The distribution of weight-bearing in the lower limbs is considered to be an important factor affecting balance. Ideally, 50% of the body weight should be handled by each of the left and right lower limbs [19, 20]. The ideal load-bearing distribution of each foot is generally considered to be 2/3 on the posterior foot [18, 19, 21]. Morasiewicz et al. evaluated the distribution of load on the lower limbs and balance before and after ankle arthrodesis with the Ilizarov method [22, 23]. They found the distraction-corrective Ilizarov corticotomy may provide more symmetric distribution of lower limb loads and improvement of balance [22, 23]. Our study found that the patients undergoing TCTD surgery still have problems of their static balance and load distributions of lower limbs. In the present study, although the weight distribution on the feet of the healthy individuals was not ideal by this definition, the mean weight handled by the posterior foot was significantly larger than that handled by the anterior foot. In the patient group, the load on the affected side was significantly smaller than that of the unaffected side. In addition, the load handled by the anterior foot was significantly larger on the affected side than that on the unaffected side, and the corresponding load on the affected side posterior foot was correspondingly smaller than that of the unaffected posterior foot. This implies an asymmetry in weight-bearing, which may have increased the degree of postural instability. Sit-to-stand training with visual feedback may represent a useful means of re-establishing balance in such patients. [19, 20, 24]

This study has following limitations. When the static balance and plantar load distribution were evaluated during the study, although we drew footprints on the floor to indicate how the participants should stand, in reality the feet of each of the participants may have been placed differently, to avoid pain in the affected foot. This may have affected the results, and represents a limitation of the study. In addition, the use of a control group that comprised a group of patients with the same pathology who had yet to undergo the same surgical procedure or who were not to undergo surgery would have increased the validity of the study. Most patients with diabetic foot ulcer cannot stand independently, and usually require crutches or a wheelchair to come to the hospital before surgery. Therefore, it is very difficult to evaluate the standing balance function of their lower extremities and their plantar load distribution before surgery. Therefore, this represents another limitation of the present study.

In conclusion, although TCTD in combination with debridement and VSD represents an alternative method for the treatment of diabetic foot ulcer, impairment in static balance and plantar load distribution remain after this procedure. The static balance is impaired mainly in the lateral plane and the plantar load is lower on the posterior foot on affected side, which should be considered when planning further rehabilitation.

## Data Availability

The datasets used and analyzed during the current study are available from the corresponding author on reasonable request.

## Abbreviations

TCTD: Tibial cortex transverse distraction
VSD: Vacuum sealing drainage
VAS: Visual analogue score
PAD: Peripheral artery lesions
COP: Center of pressure
